# Bladder response to acute sacral neuromodulation while treating rats in different phases of complete spinal cord injury: a preliminary study

**DOI:** 10.1590/S1677-5538.IBJU.2014.0144

**Published:** 2015

**Authors:** Ping Shi, Youfang Fang, Hongliu Yu

**Affiliations:** 1 Institute of Rehabilitation Engineering and Technology - University of Shanghai for Science and Technology, Shanghai, China

**Keywords:** Spinal Cord Injuries, Urinary Bladder, Urinary Incontinence

## Abstract

**Background:**

Compared to conventional therapies, sacral neuromodulation (SNM) may offer an alternative, non-destructive treatment for SCI patients with bladder dysfunction. Understanding bladder response to SNM treatment for SCI in different phases may yield new insights for innovative use of this promising technique.

**Materials and Methods:**

Female Sprague-Dawley rats were used in this study to examine the effects of acute SNM on bladder reflex in complete SCI rats. All rats were anesthetized and set up for continuous saline infusion. Acute SNM treatment was implemented for about 6 hours for each rat. Cystometric parameters, including time between contractions, contraction duration, bladder peak pressure, and number of uninhibited contractions, were analyzed and compared within rats before and after SNM treatment.

**Results:**

For the spinally transected rats during early phase (less than two weeks post spinalization), the time between contractions and contraction duration both increased after SNM treatments, yet the increased amplitude was about or less than 20%. For the spinally transected rats with a longer days survival (about two to four weeks post spinalization), the time between contractions and contraction duration substantially increased after SNM treatment and the changes for their average values were more than 90%. For the spinally transected rats with a much longer days survival (more than five weeks post spinalization), the time between contractions and contraction duration increased after SNM treatments, yet the magnitude of changes were less than 30%.

**Conclusion:**

The present study suggested that the significant effectiveness of SNM for complete SCI played its role after the spinal shock phase and prior to the development of detrusor overactivity. It indicated that the time point of SNM treatment is necessary to be paid attention.

## INTRODUCTION

Supra-sacral lesions to the spinal cord nearly always lead to serious disruption of lower urinary tract function (LUTD). Previous reports showed electrical stimulation has emerged as a valuable minimally invasive treatment option for patients with LUTD in whom conservative treatments have failed. The locations of stimulation used in patients with supra-sacral spinal cord injury (SCI) or disease have been reported in a number of ways, including the bladder wall (1), the pudendal or dorsal genital nerve (2, 3), the conus medullaris (4), the tibial nerve (5), the sacral anterior roots (6), or the mixed sacral nerves (7, 8). In practice, only the latter two sites have demonstrated clinical significance. However, stimulation of the sacral anterior roots always combined with posterior sacral rhizotomy can prevent many suitable patients from accepting this therapy, although cystometry and clinical examination should show that rhizotomy is effective to suppress reflex incontinence.

Sacral neuromodulation (SNM) may be an alternative solution to sacral deafferentiation, which involves stimulation of sacral afferent pathways rather than cutting them to suppress reflex incontinence (8–10). From early application of SNM until now continuous research is carried out to improve this therapy and to determine the exact mechanism of action. The efficacy of SNM for treatment of LUTD probably relies on spinal and supra-spinal reflex arcs (11). This assumption is supported by the observation that SNM is not effective in patients with complete or nearly complete SCI (12, 13). Recently, Sievert and colleagues's investigation indicated that early SNM in patients with complete spinal cord injury during spinal shock (ie, the bladder arreflexia phase) prevented detrusor overactivity and urinary incontinence (14). Prevention of LUTD before irreversible effects occur is a convincing concept and the findings reported by Sievert and colleagues are exciting. Their research emphasized the significance of the time point of SNM.

Taking into account that SNM is minimally invasive and completely reversible, it is of great interest whether this treatment option is valuable for neurogenic LUTD following complete SCI before resorting to more invasive procedures. Our previous study (15) have substantiated that SNM could offer an alternative, non-destructive treatment for complete SCI animal with bladder dysfunction about three weeks post-spinalization to resemble the condition of urinary bladder hyperreflexia. Further, it is necessary to examine the effect of SNM implementation during different phases after spinalization.

The goal of this study is to investigate the effects of acute SNM on the bladder responses in model rats with complete spinal cord lesion after different days of model surgery.

## MATERIAL AND METHODS

### Animal Model of SCI

All animal care and experimental procedures were reviewed and approved by the Institutional Animal Care and Use Committees of Shanghai University for Science and Technology. Experiments were performed on female Sprague-Dawley (250g-300g) rats. In order to create an urodynamic pattern similar to humans with supra-sacral SCI, T9-T10 level were considered by researchers to be the spinal transection site for the experiment rats (16).

Under general anesthesia with chloral hydrate (400mg/kg), the rat underwent complete spinal cord transection by a micro-scissor after laminectomy at the T9-T10 level. The rat's body temperature was maintained at 37ºC during and after the surgery using a heating blanket until it woke up. To ensure complete disconnection of spinal fibers, the open cavity separating the two ends was filled with hemostatic gel foam. The muscle and skin were then sutured. The animal was returned to its cage after full recovery from anesthesia. Upon awakening from anesthesia, animals were given buprenorphine (0.1mL/100g body weight) subcutaneously for pain control. Postoperatively, rats were housed in shallow cages with high absorbent bedding and had access to food and water ad libitum. Penicillin (15–20mg/kg sc) was administered once daily during the feeding. The bladder was manually expressed twice daily (Crede's maneuver).

### Cystometric Studies

The spinally transected rats (7, 12, 15, 18, 20, 27, 36 and 42 days survival, respectively) were anesthetized with chloral hydrate (400mg/kg).

Cystometric study was performed using transvesical catheter implanted into the bladder dome. Transvesical ways, although more invasive than a transurethral way, prevent the impossibility of the cystometric recording because the external urethral sphincter closes tightly following complete SCI. Therefore, the leaking urine was not detected in this study since the external urethral sphincter closes tightly. Before cystometric study, the bladders were manually expressed using Crede's maneuver. After a midline abdominal incision, the urinary bladder was exposed and a polyethylene tube (PE-60, 1.0mmID and 1.5mm OD) was inserted into the dome of the bladder. The free end of the implanted catheter was connected via a T-stopcock to a pressure transducer (MLT0380/D, ADInstruments Pty Ltd, Sydney, Australia) for monitoring bladder pressure and an infusion pump for infusion saline. The tube was secured with a purse-string suture and the incisions were closed in layers.

**Figure 1:**
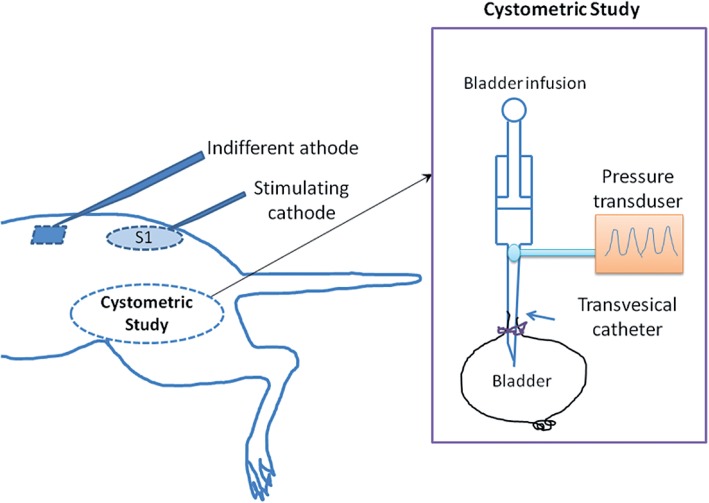
Schematic diagram of the experimental set up on the rats for recording bladder activity with SNM treatment. A PE tube was inserted into the bladder dome, which was in turn connected via a three-way stopcock to an infusion pump for filling with saline, and to a pressure transducer for monitoring bladder pressure. The cathode electrode was inserted into the S1 foramina, and the anode was placed under the skin of the back.

During surgery, animal body temperature was maintained at 37°C using a heating blanket. Room temperature saline solution was infused continuously into the bladder with catheter. The intravesical pressure signal was stored using a biological signal collecting and processing system (PowerLab 4/26, AD Instruments Pty Ltd, Sydney, Australia). Urodynamic characteristics of bladder contractions were investigated in this study. Several parameters were calculated based on urodynamic signal, including bladder contraction duration, bladder contraction cycle period (the time interval between two continuous contractions), peak bladder pressure and the number of uninhibited contraction. The cystometric parameters were recorded before and after SNM treatment. The number of uninhibited contractions was also recorded. The experimental setup is shown in Figure-1.

### SNM Treatment

Unilateral sacral foramen electrode has been the gold standard for SNM (17). Indeed, there is no evidence that bilateral simultaneous stimulation has any added benefits to unilateral stimulation (18). Unilateral sacral segmental stimulation with an electrode at the level of the sacral foramen S1 was accepted and performed by most researchers in the rat with SNM experiment (19, 20). In the experiment with acute SNM, the time of SNM treatment was not the same between experiments, however, a short or a long treatment time was considered to be inefficient or lead to increased mortality in experimental animals. Therefore, in this study the unilateral S1 roots of rat were electrically stimulated for 6 hours using stainless steel electrodes inserted into the S1 foramina.

**Figure 2:**
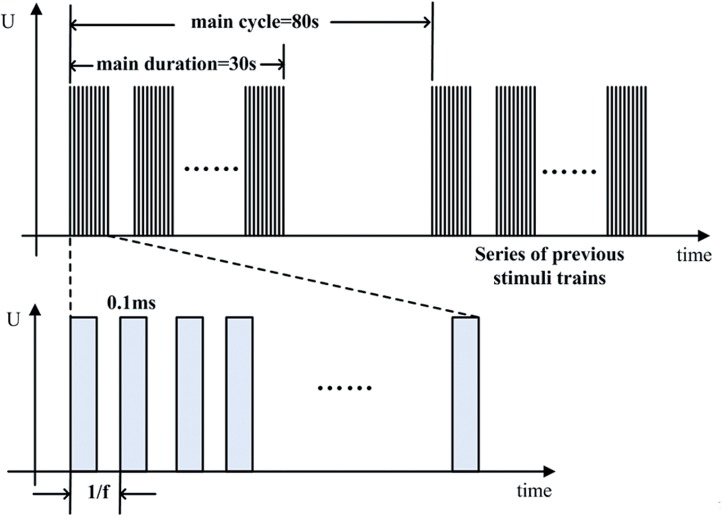
The stimuli used in the experiments. The pulses were monophasic negative pulses with frequency of 20 Hz, pulse duration of 0.1ms, train duration of 30 sec, and train period of 80 sec. The stimulation amplitude was about 1.5–4.0 V, which is variable for individual rats.

The stimuli used in the experiments were monophasic negative pulses with frequency of 20Hz, pulse duration of 0.1ms, train duration of 30 sec, and train period of 80 sec (Figure-2). The stimulation amplitude was adjusted to 80% of the value that induced a visible tail tremor (about 1.5–4.0 V, which is variable for individual rat). Before and after stimulation, the rats underwent continuous urodynamic recording with saline infusion at the rate of 0.1mL/min. During the period of experiment study, analgesic depth was assessed continuously by the eyelash reflex and the paw retraction on moderate pinching. Anesthesia was maintained with low dose of chloral hydrate (100mg/kg).

### Data Analysis

In the study, 20 values of the cystometric parameters were applied to analysis. Therefore, 20 cycles of bladder response for each rat before and after SNM treatment were recorded. The intravesical pressure was recorded and analyzed by two different persons, and the latter was blinded to the rat's conditions. The results are expressed as mean and standard deviation (±SD). The significance of differences before and after SNM treatment was compared. A P<0.05 was considered statistically significant. The statistical analyses were run in MATLAB software (Math Works Inc., MA, USA).

## RESULTS

**Figure 3:**
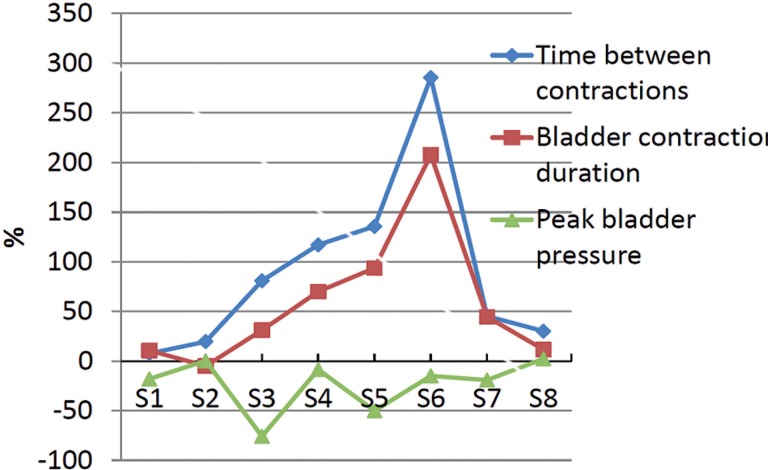
Changes of average values of parameters after SNM treatment. The parameters, i.e. time between contractions and bladder contraction duration, for the SCI rat in the late phase of spinal shock or shortly after the spinal shock phase present a substantial increase.

SCI rats in different phases were treated with SNM. Cystometric parameters before and after SNM treatment, including contraction cycle period, contraction duration, bladder peak pressure, and number of uninhibited contractions, are presented in Table-1. The SCI rats were analyzed individually with bladder infusion because the condition of rats and the parameters used in electrical stimulation are not identical, and it is also necessary to evaluate the effect of the therapeutic neuromodulation for each rat. For the spinally transected rats during early phase less than two weeks post spinalization, i.e. within 7 and 12 days survival in the present study, the time between contractions and contraction duration both increased after SNM treatments (Table-1), yet the increased amplitude was about or less than 20% (Figure-3). The peak bladder pressure decreased or had little changes. For the spinally transected rats with longer survival days (about two to four weeks post spinalization, i.e. with 15, 18, 20 and 27 days survival in the present study), the time between contractions and contraction duration showed substantial increases after SNM treatment (Figure-3). Especially, the parameters of time between contractions and contraction duration for SCI rats with 20 and 27 days survival dramatically increased (P<0.05, Table-1) and the changes for their average value were more than 90% (Figure-3). For the spinally transected rats with more than five weeks post spinalization, i.e. 36 and 42 days survival in the present study, the time between contractions and contraction duration increased after SNM treatments (Table-1), yet the magnitude of changes were about or less than 45% (Figure-3). The parameter of peak bladder pressure decreased or changed little after SNM treatment (Figure-3). The uninhibited contraction appeared during the late phase of SCI, and its number decreased after the SNM treatment (Table-1). Due to the high mortality rate associated with lower urinary tract complications and decubitus ulcers infection, the details of the parameters for SCI rats with a very long survival days have not been included.

**Table 1:** Measurement from the SCI rat 7, 12, 15, 18, 20, 27, 36 and 42 days post surgery before and after SNM treatment under saline infusion with 0.1mL/min. bSNM: before Sacral Neuromodulation; aSNM: after Sacral Neuromodulation.

Rat	Conditions	Time	Time between contractions (s)	Bladder contraction duration (s)	Peak bladder pressure(cm H_2_O)	No. of uninhibited contraction
S1	7 days post surgery	bSNM	68.5±50.5	49.5±43.6	3.7±1.3	0
		aSNM	73.8±36.7	54.8±22.7	3.0±0.4	0
S2	12days post surgery	bSNM	48.6±35.5	39.4±32.8	3.9±1.7	0
		aSNM	58.2±47.0	37.5±14.6	3.9±1.8	0
S3	15 days post surgery	bSNM	20.9±9.3	19.7±8.7	11.3±6.7	0
		aSNM	37.9±39.4	25.8±25.1	2.7±1.3	0
S4	18 days post surgery	bSNM	79.1±53.0	35.0±17.3	3.4±1.4	0
		aSNM	171.7±156.9	59.5±38.6	3.1±1.5	0
S5	20 days post surgery	bSNM	62.0±18.1	30.3±4.5	7.7±5.5	2
		aSNM	146.2±137.3*	58.6±19.8*	3.9±1.4	0
S6	27 days post surgery	bSNM	24.9±6.5	22.5±5.9	7.0±3.2	1
		aSNM	96.2±31.7*	69.31±23.88*	6.0±2.7	0
S7	36 days post surgery	bSNM	52.2±29.7	28.3±20.1	11.8±5.2	10
		aSNM	75.8±31.6	40.9±31.6	9.6±7.0	8
S8	42 days post surgery	bSNM	40.4±16.5	33.7±10.3	20.9±13.9	17
		aSNM	52.6±43.2	37.6±26.0	21.4±14.6	12

* P<0.05 vs bSNM

## DISCUSSION

The control of the lower urinary tract is a complex, multilevel process that involves both the peripheral and central nervous systems (21). The lesion of spinal cord produces LUTD by eliminating the brain mechanisms. SNM has become a well-established and widely accepted treatment modality in recent years for LUTD (22), whereas SNM has been attempted without success in complete SCI patients (13). However, a recent study (14) in which bilateral SNM was initiated early during the recovery period from complete thoracic spinal cord injury prevented the development of neurogenic detrusor overactivity and urinary incontinence. It is assumed that the effectiveness of the SNM treatment to some extent was determined by the dynamic neurologic process and reorganization or neuroplasticity that might occur after a spinal lesion, emphasizing the significance of the time point of SNM treatment. In the present study, SNM was treated on completely spinalized rats to understand its effectiveness via urodynamic parameters during different SCI phases.

With SCI the normal connections between the sacral cord and the supraspinal circuits that control urine storage and release are disrupted. Patients or animals in the early phase of SCI, e.g. spinal shock phase, will typically present with overflow incontinence due to detrusor failure or severe bladder outlet obstruction (15). Experimental studies in rats have shown that, soon after SCI, bulbospinal pathways are damaged and this disrupts the control of sympathetic preganglionic neurons (23). In the present study, SNM treated during spinal shock phase (rats S1, S2, S3 and S4) didn't achieve a significant result. However, it is worth mentioning that during the late phase of spinal shock phase (rat S4) the treatment of SNM improved the bladder function including significant promotions of time between contractions and contraction duration. This phenomenon seems to imply the neuromodulation of the electricity current initially play its part in the late spinal shock phase during which the reflex neurogenic bladder and autonomic hyperreflexia appears (24). During late spinal shock phase, electrical stimulation influences spinal cord plasticity and performance including promoting synaptic maturation and refinement of neural circuits (24).

After the spinal shock phase, detrusor overactivity develops (15). This overactivity is mediated by a spinal micturition reflex that emerges in response to a reorganization of synaptic connections in the spinal cord (25). During this phase, C-fiber bladder afferents proliferate within the urothelium and become sensitive to bladder distention. These changes lead to the emergence of a new C-fiber-mediated voiding reflex, which is strongly involved in detrusor hyperreflexia (26). In the present and our previous study (2), the significant effectiveness of SNM is observed during this phase of SCI. Sievert and colleagues indicate that early SNM may preserve nerve plasticity, such that C-fibres remain silent, detrusor overactivity is avoided, and sympathetic preganglionic neuron activation in the thoracolumbar cord is suppressed, supporting detrusor contractility (14). The precise mode of action of SNM is still unclear but it has been hypothesized that the electrical current modulates reflex pathways involved in the filling and evacuation phase of the micturition cycle (27). However, supra-spinal pathways seem to be involved as well (28). Studies in animals indicate that dysfunction of the lower urinary tract after SCI is dependent in part on plasticity of bladder afferent pathways as well as reorganization of synaptic connections in the spinal cord. Bladder afferent nerves are critical for sending signals of bladder fullness and discomfort to the brain and for initiating the micturition reflex. Over a period of several weeks following cord injury, major neuroplasticity appears within bladder afferent circuitry. Researchers conclude that one of mechanisms of SNM treatment is to influence the spinal neuronal circuits via the activation of afferent nerve fibers causing inhibition of the voiding reflex at a spinal and/or supraspinal level (29). The effectiveness of SNM from the clearly evident improvement in urodynamic parameters in the present results indicate that SNM prevent the reorganization of afferent nerve fibers, e.g. bladder C-fiber afferents that contributes to detrusor overactivity. Also, development of detrusor overactivity is related to the interrupted regulatory mechanism between the lower urinary tract and midbrain for urine storage and voiding (30), suggesting the SNM works through the remaining intact sympathetic trunk extending to the brain (bypassing the SCI).

In the results from the present study, the efficacy of SNM substantially weakened about five weeks post spinalization although the number of uninhibited contraction decreased due to SNM probably. Also in clinical practice, the biggest obstacle to the acceptance of SNM is its potential to fail over time. The reasons for these failures are not clear, but the natural plasticity of the nervous system, leading to reactivation of pathological reflex arcs (31), was considered one of possible explanations.

## CONCLUSION

Electrical stimulation has been investigated for many years for the purpose of restoring function to the neurogenic bladder. SNM for treating functional voiding dysfunction has become established in urology. SNM was originally not considered an option for neurogenic LUTD, and SNM has been attempted without success in complete chronic SCI patients. The present study indicated that the time point of SNM treatment is necessary to be paid attention. Although SNM seems to be a promising therapy for neurogenic disease, further studies and long-term results with an extended cohort of complete SCI patients are yet to be obtained.
